# A Good Test for Coffee

**Published:** 1890-02

**Authors:** 


### A GOOD TEST FOR COFFEE.

Spread a few grains on a glass plate and moisten them with a little water, when any foreign vegetable substance will swell and become soft, while the coffee remains hard. Genuine coffee will float on the surface of cold water for a considerable time, and will be hours imparting much color to it. If you want to see this test, buy a pound of sixteen-cent, ready-ground coffee and a pound of the whole article sold at thirty-five cents. Have the latter ground and perform the test of each of the two kinds in separate glasses. In twenty minutes or a half hour the water in the glass containing the low-priced article will be markedly colored, and chicory and other foreign vegetable matter will have sunk to the bottom of the glass; while the water of the other sample will be but slightly if at all discolored, and a little or none of the coffee placed on the surface will have sunk. Care should be taken not to shake the glasses during the test. This experiment is practical, interesting and convincing.

